# Humans in a Dish: The Potential of Organoids in Modeling Immunity and Infectious Diseases

**DOI:** 10.3389/fmicb.2017.02402

**Published:** 2017-12-05

**Authors:** Nino Iakobachvili, Peter J. Peters

**Affiliations:** Division of Nanoscopy, Maastricht Multimodal Molecular Imaging Institute, Maastricht University, Maastricht, Netherlands

**Keywords:** organoids, disease model, infection, macrophages, monocytes, dendritic cells, MPS, tuberculosis

## Abstract

For many decades, human infectious diseases have been studied in immortalized cell lines, isolated primary cells from blood and a range of animal hosts. This research has been of fundamental importance in advancing our understanding of host and pathogen responses but remains limited by the absence of multicellular context and inherent differences in animal immune systems that result in altered immune responses. Recent developments in stem cell biology have led to the *in vitro* growth of organoids that faithfully recapitulate a variety of human tissues including lung, intestine and brain amongst many others. Organoids are derived from human stem cells and retain the genomic background, cellular organization and functionality of their tissue of origin. Thus they have been widely used to characterize stem cell development, numerous cancers and genetic diseases. We believe organoid technology can be harnessed to study host–pathogen interactions resulting in a more physiologically relevant model that yields more predictive data of human infectious diseases than current systems. Here, we highlight recent work and discuss the potential of human stem cell-derived organoids in studying infectious diseases and immunity.

## Introduction

Lower respiratory infections, cancers (including those caused by infectious agents), diarrheal diseases and tuberculosis remain among the top 10 causes of death worldwide (WHO, 2017). Of crucial importance in sustaining organismal homeostasis, coordinating defenses against pathogenic attack and orchestrating the innate and adaptive immune responses is the mononuclear phagocyte system (MPS)- a key network of macrophages, dendritic cells and monocytes. The tissue resident macrophages function to clear cell debris, resolve inflammation and modulate immune responses (Davies et al., 2013); antigen-presenting dendritic cells initiate the adaptive immune response and prime the immune system to future attack (Mildner and Jung, 2014); and lastly, circulating monocytes migrate around the body where they can remain as monocytes, differentiate into macrophages in response to stimulatory signals or acquire antigen presenting abilities (Chow et al., 2011). The human MPS is part of the first line of immune defense, however, modeling this complex system in relation to infection, disease and development is difficult and is predominantly performed using three strategies:

(1) Purification of peripheral blood mononuclear cells (PBMC’s) from human blood for short term *in vitro* culture (with the possibility to differentiate the monocytes to macrophages or dendritic cells); similarly, but in lower numbers, dendritic cells can be purified straight from blood or tissue resident macrophages from certain tissues, e.g., alveolar macrophages from lungs,
(2) Immortalized MPS-like single cell lines of human or animal origin, e.g., the human monocyte-like THP-1 cell line, the murine macrophage-like J774 cell line,
(3) Using animal organisms to study aspects of the system, e.g., non-human primates, zebrafish, mice, humanized mice, cell lineage depleted animals and extrapolating results to humans.

It is key to note that single cell culture and animal models are widely used and have been instrumental in many scientific advancements in fundamental research, drug and vaccine development, infectious, autoimmune and genetic diseases to name a few. Whilst their scientific importance cannot be underestimated, critical questions typically arise relating to the validity of these systems. This is a result of the inherent differences in cellular context (or the lack of it when using primary cells or cell lines), physiology and genetics of different species which influences disease outcome, progression and accurate prediction of human response. The severe effects of such matters become apparent in the fact that pre-clinical animal tests are still failing to predict human pharmacodynamics and toxicity as exemplified by the recent failure of the 2016 phase 1 clinical trial of BIA 10-2474 for neuropathic pain which resulted in the hospitalization of five participants and the death of one (Chaikin, 2017). With an estimated 90% of drugs that pass pre-clinical tests failing in human clinical trials (Mullard, 2016), it is not surprising that the scientific community is increasingly encouraging the development and exploitation of alternative approaches that may offer a more valid way of modeling diseases, disorders and drug interactions (Korch et al., 2011). In addition to the questionable validity of these non-human systems, the guiding principles of the ‘3 R’s of animal research’ have been increasingly implemented in new legislation where animals are being used for research (United States, Canada, United Kingdom, and Europe). The 3 R principles, a set of ethical guidelines first published in Russell and Burch (1959) aim to Replace the use of animals for scientific research, Reduce the number of animals used and Refine techniques to minimize the pain, suffering and distress caused to animals during scientific research. Consequently the need for human *in vitro* based systems is at a record high. We believe that the future of applied and fundamental research lies in the quickly expanding field of *in vitro* grown organoids that recapitulate human organs to varying degrees. Organoids bridge the gap between single cell culture and *in vivo* work, offering ethically obtained functional, multicellular tissue of human origin that provides a more similar *in vitro* system in which to study multiple components of host–pathogen interactions and drug response.

## The Organoid Revolution

**FIGURE 1:**
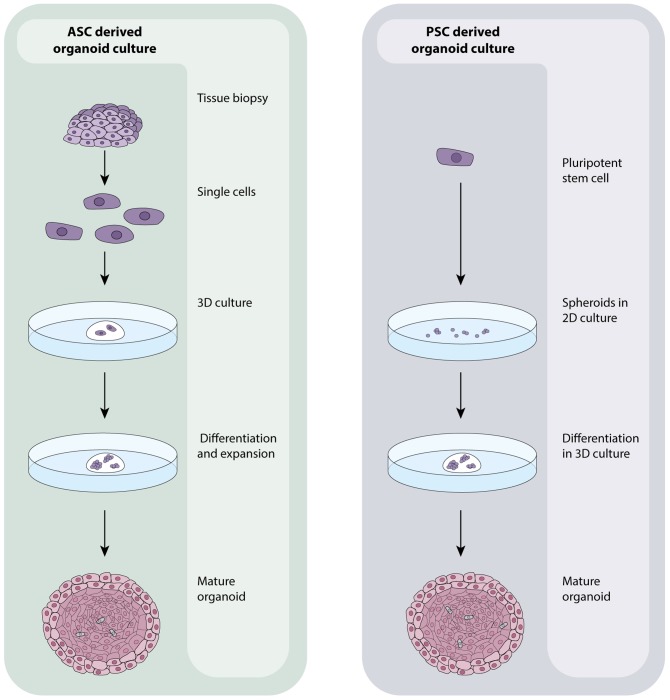
ASC- and PSC-derived organoid culture protocols. ASC-derived organoids are grown from healthy or tumourous tissue biopsies. Tissues are processed into a single cell suspension which is directly embedded in an extracellular matrix. Media containing numerous growth factors, which vary dependent on the tissue being grown, is added and regularly changed until organoids have expanded. PSC-derived organoids originate with 2D cultures of PSC’s that are grown into aggregates/spheroids. At this point, they may be embedded in an extracellular matrix and expanded to maturity using a a growth factor rich media that is again, specific to the tissue of interest. The composition and organization of cells within organoids is dictated by the identity of the tissue being grown which in turn affects the overall size and shape of the organoid.

Recent advances in stem cell biology have allowed researchers to grow human tissues that resemble organs *in vitro*. These organoids are stem cell derived, self –organizing, multi-cellular aggregates that closely recapitulate the function, cellular components and architecture of human tissues. Organoids are cultured in 3D (in extracellular matrix) and reflect the cellular heterogeneity and cellular behavior of tissues *in vitro*. Unlike cell lines, organoids remain genetically stable for long periods (years) and do not show significant increases in the expression of stress-related genes during extended culture (Sato et al., 2009). In addition to their close resemblance to human tissues, organoids are amenable to the same analytical techniques as primary cells/cell lines including fluorescence labeling, live cell imaging, electron microscopy, mass spectrometry (Cristobal et al., 2017) and genetic manipulation including by CRISPR/Cas9 (Matano et al., 2015) making them particularly suitable for scientific research. Typically, organoids are derived from either embryonic or induced pluripotent stem cells (PSC’s) or adult stem cells (ASC’s) resected or biopsied from organs. Both cell types have the unique capacity to self-renew and differentiate- a defining feature of stem cells, and the absence of which in primary cells causes their quick death *in vitro* (Melton, 2014). Organoids derived from ASC’s are grown from cell suspensions obtained from primary tissue biopsies or resected material that are immediately embedded in an extracellular matrix and grown in the presence of specific growth factors to direct cell differentiation (**Figure 1**). Organoids that can be cultured from human ASC’s include colon (Sato et al., 2011), intestine (Sato et al., 2009), liver (Huch et al., 2013b), prostate (Karthaus et al., 2014), pancreas (Huch et al., 2013a), fallopian tube (Kessler et al., 2015), stomach (Barker et al., 2010), tongue (Hisha et al., 2013) and endometrium (Turco et al., 2017). Those derived from PSC’s are generally grown from 2D cultures of stem cells that are matured into spheroids, committed to endoderm using Activin A (Takebe et al., 2013) and then cultured in 3D with specific differentiation signals that are dependent on the type of tissue that is ultimately required (**Figure 1**). Intestinal (Spence et al., 2011), liver (Takebe et al., 2013), lung (Dye et al., 2015), kidney (Takasato et al., 2015), pancreas (Huang et al., 2015), stomach (McCracken et al., 2014), and retinal (Völkner et al., 2016) organoids can all be cultured from PSC’s. PSC-derived organoids that are not derived from endoderm can also be cultured with modifications to the above protocol as exemplified by cerebral (Lancaster et al., 2014), optic-cup (Eiraku et al., 2011) and kidney (Takasato et al., 2015) organoids.

The similarity of some organoids to their tissue of origin is exemplified in elegant research by the Clevers laboratory who first identified the Lgr5+ intestinal stem cell (Barker et al., 2007) and then characterized the signals (epidermal growth factor, Wnt-3, R-spondin and Noggin) required for maintaining these cells in culture and promoting their proliferation and cellular differentiation into intestinal epithelium (Sato and Clevers, 2013). This research established the first ASC-derived organoids, and has formed the basis for the growth cocktails used to culture a variety of different mouse and human tissues including GFP expressing colonic mouse organoids. When GFP+ colon organoids are transplanted into mice treated with colitis-inducing dextran sulfate sodium, intestinal lesions show signs of recovery within 16 days of transplantation. Transplanted organoids were histologically indistinguishable from the surrounding epithelium, fully functional, contained all terminally differentiated cell types and recovered the body weight of diseased mice (Yui et al., 2012).

This pioneering method of tissue culture has thus unlocked an entirely new tool that can be harnessed in scientific research- one that provides a consistent, genetically stable source of multicellular human or animal tissue for experimental use. Additionally, whilst PSC-derived organoids typically recapitulate early stages of cellular proliferation that can be used to study development and foetal infections for example, ASC derived organoids can provide complimentary, adult epithelium to explore mature tissue responses to pathogenic attack or drugs. Thus developmental biologists have already begun to use organoids to study organogenesis (Shyer et al., 2015), lineage specification (Yin et al., 2014), stem cell niche and tissue homeostasis (Barker, 2013). Organoids can also be grown from healthy and tumorous human tissues (Van De Wetering et al., 2015)- in both cases, retaining some, if not most, of the features of the original tissue. Thus cancer biologists are extensively modeling *in vitro* patient heterogeneity (Weeber et al., 2015), metastatic potential (Nadauld et al., 2014) and drug screening. Due to their genetic and morphological similarities to the organ from which they are derived from, ASC organoids provide a novel approach to study stem cell and tissue transplantation. This feature is a key factor in making organoid-based personalized medicine a reality and which is exemplified by Beekman and colleagues who have applied intestinal organoids grown from cystic fibrosis (CF) patients to screen for drugs. Specifically for those that restore the function of mutant CFTR (an anion channel called CF transmembrane conductance regulator that, when mutated, is responsible for causing CF in approximately 67% of cases worldwide) proteins (Dekkers et al., 2012). Considering the difficulties in predicting patient responses to drugs, and the expense of providing drugs that are ultimately inefficient, the development of this assay is an important step toward facilitating diagnosis, drug development and personalized treatment regimens. This discovery has been successfully translated to the clinical setting (Saini, 2016). It is clear that organoid systems have already begun to be widely and successfully used in a range of clinical and basic research environments and naturally, the infectious diseases community has also taken notice.

## Organoids in Infectious Diseases

Zika virus infection (ZIKV) of humans was first identified in Dick et al. (1952); it is spread by mosquitoes and typically illness is mild with fever-like symptoms for 2–7 days. In 2016, ZIKV was declared a public health emergency by WHO based on epidemiological evidence that there was an association between babies born with neurological complications including microcephaly and ZIKV. At this point, no suitable model existed for studying this pathogen but microcephaly had been successfully modeled in induced PSC-derived cerebral organoids that develop discrete brain regions (Lancaster et al., 2014). ZIKV was modeled in such a system to great success; infection was found to cause neuronal cell death in the early stages of brain development (Garcez et al., 2016), to induce premature differentiation of preferentially infected neural progenitor cells resulting in mitotic defects (Gabriel et al., 2017), and to upregulate the innate immune receptor TLR3 (toll-like receptor 3) which resulted in dysregulation of neurogenesis and cell death (Dang et al., 2016). The organoid model of ZIKV has also been adapted to a large scale platform that can be used for modeling brain development, neurological diseases and drug screening by growing brain region specific organoids in miniaturized spinning bioreactors (Qian et al., 2016) further highlighting the power and success of using organoids for studying development, disease and treatment. Organoids have also proved themselves to be a robust, accurate and reproducible model to study Norovirus (Ettayebi et al., 2016), and clinical strains of Rotavirus (Finkbeiner et al., 2012; Zhu et al., 2017)- two viruses that were difficult to cultivate and study *in vitro* until organoids became available as a tool.

**FIGURE 2:**
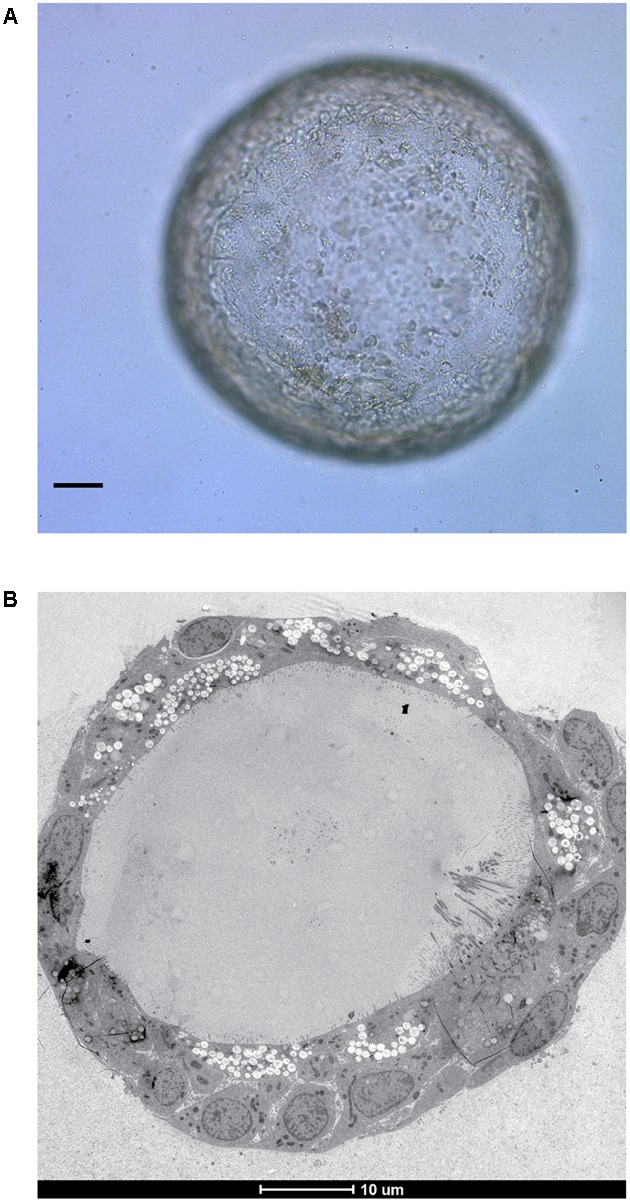
*In vitro* grown ASC-derived lung organoid. **(A)** Mature lung organoid in extracellular matrix on day 25 of culture. Scale bar is 10 μm. **(B)** Electron microscope image of a mature lung organoid following high pressure freezing, freeze substitution, epon embedding and sectioning. Note self-organization of multiple differentiated cells. Both courtesy of Norman Sachs.

Bacterial pathogens are also being increasingly studied in organoids. Organoid based studies into *Salmonella enterica* serovar Typhi virulence provided the first evidence that infection with this bacteria is a causative agent of gallbladder carcinoma (Scanu et al., 2015). *S.* Typhimurium was found to alter the organoid transcriptome to activate the Akt and MapK kinase pathways which are often found to be elevated in human cancers (Manning and Cantley, 2007; Cseh et al., 2014). Similar features were presented in mouse gallbladder carcinomas and infected gallbladder organoids including loss of cellular polarity and enlarged nuclei. Interestingly, cells dissociated from organoids with previous *S.* Typhimurium infection were able to expand into new organoids in growth factor-diminished media, unlike those which had not been previously exposed to infection indicating a sustained alteration of host signaling pathways. PSC-derived intestinal organoids are being used to study shigatoxigenic serotypes of *Escherichia coli* for which no suitable animal models exist (Karve et al., 2017), whilst gastric organoids from ASC’s are being developed as a model in which to study *Helicobacter pylori* infection (Bartfeld et al., 2015; Schlaermann et al., 2016) – with potential to extend the model to encompass the study of *H. pylori* associated gastric cancer. Our own laboratory is focused on modeling tuberculosis infection within ASC-derived lung organoids (**Figure 2**) to continue our research on unraveling the mechanisms behind ESX-1 dependent translocation and its function during infection (van der Wel et al., 2007; Houben et al., 2012).

Studying interactions between human hosts and their protozoan parasites has been difficult due to a lack of appropriate animal models or due to the difficulty in culturing parasites with obligate human-host specificity. Currently, organoids have been limited to a supportive role to assay the role of tuft cells during infection with the helminth parasite *Nippostrongylus brasiliensis* (Gerbe et al., 2016). However, this field recognizes the ability of organoid technology in overcoming the experimental bottlenecks described above to model parasitic pathogens such as *Cyclospora* sp., *Cryptosporidium* sp. and *Giardia* sp. (Klotz et al., 2012).

## Increasing the Complexity of Organoid Systems

It is clear that organoids can represent tissue structure and function exceptionally well. Organoid technology is a significant and brilliant advancement in the tools available for scientific research across a wide range of different topics but they remain inherently incomplete- they lack the microenvironment of stroma, vasculature, immune cells and other organ systems that tissues interact with in the body during development and disease. It is also likely that not all developmental stages are currently being represented in a single organoid culture which is particularly relevant if fetal or adult infections are being modeled. Furthermore, certain cell types are not being maintained in organoid cultures due to incorrect signaling resulting in the growth of ‘incomplete’ tissues that compromise the validity of these systems in research.

Organoid culture techniques are thus in a constant state of evolution; culture conditions are constantly being improved to better support multiple cell types and drive proliferation. Current efforts are also guiding the development of designer matrices such as polyethylene glycol hydrogels that are mechanically more stable and whose composition is defined, unlike the inherently variable, animal sourced and traditionally used extracellular matrix (Gjorevski et al., 2016). Better defined culture conditions are more conducive to controlled modifications and will render organoids more accessible in clinical and basic research where reproducibility is important. Efforts are also being made to address the lack of vascularization in organoid cultures to improve nutrient availability, signaling and removal of toxins as would occur naturally in the body thus bringing organoids one step closer toward functional, *in vitro* grown organs. This is currently being accomplished by transplanting induced PSC-derived organoids grown with stromal populations and connecting the endothelial network to a mouse host (Takebe et al., 2013; Raikwar et al., 2015), however, it remains labor intensive and does not yet translate easily to standard tissue culture facilities.

Most important for studying the MPS in organoids, are advancements relating to co-culturing organoids with cell types that are absent in epithelial structures such as macrophages and dendritic cells. Though currently in its infancy, it is already possible to mix intestinal organoids cultured as normal, with purified cultures of intraepithelial lymphocytes in an extracellular matrix to study their migration against the basal side of the organoids via fluorescent imaging (Nozaki et al., 2016). It is also possible to microinject cells of interest into the lumen of organoids to ensure contact with the apical side if exogenously added cells are unable to migrate through organoid tissue. Such co-cultures are likely to be short term due to the current short life-span of primary purified immune cells but they lead the way toward creating organoid cultures with increased complexity that allow us to study human physiology *in vitro* at an unprecedented level and in a experimentally controllable environment. Immune cells are often the first point of contact during infection, and their response dictates disease outcome. It is thus important to use organoid systems to their full capacity. Therefore, to accurately model both the MPS and infectious disease, we must be sure to include missing but key cell types, even if they are not naturally present using current methods of organoid culture.

## Concluding Remarks

The beauty of organoids in recapitulating human tissue and function *in vitro* has been described, and their use in stem cell development, cancer, infectious diseases, drug screening and transplantation demonstrated. As we get better insight into the culture conditions needed to maintain the multiple cell types and microenvironment associated with human tissues, we gain from being able to recapitulate more of human physiology *in vitro*. This in turn provides an increasingly suitable and better defined model system for modeling the role of MPS and other cells involved in infection, immunity and inflammation. Organoid systems are also exquisitely suitable for adaptation into diagnostic and screening platforms as they offer an excellent source of readily available human tissue that accurately reflects human responses. Whilst organoids are not without their caveats, we believe they will continue to significantly advance scientific attitudes, fundamental, therapeutic and clinical research for decades more.

## Author Contributions

NI conceived the idea, wrote and edited the manuscript. PP contributed ideas and discussion of the manuscript.

## Conflict of Interest Statement

The authors declare that the research was conducted in the absence of any commercial or financial relationships that could be construed as a potential conflict of interest.
